# Zebrafish Caudal Haematopoietic Embryonic Stromal Tissue (CHEST) Cells Support Haematopoiesis

**DOI:** 10.1038/srep44644

**Published:** 2017-03-16

**Authors:** Anja Wolf, Julian Aggio, Clyde Campbell, Francis Wright, Gabriel Marquez, David Traver, David L. Stachura

**Affiliations:** 1 California State University, Chico, Department of Biological Sciences, Chico, CA, 95929, USA; 2 Department of Cellular and Molecular Medicine, University of California at San Diego School of Medicine, La Jolla, CA, 92093, USA

## Abstract

Haematopoiesis is an essential process in early vertebrate development that occurs in different distinct spatial locations in the embryo that shift over time. These different sites have distinct functions: in some anatomical locations specific hematopoietic stem and progenitor cells (HSPCs) are generated *de novo*. In others, HSPCs expand. HSPCs differentiate and renew in other locations, ensuring homeostatic maintenance. These niches primarily control haematopoiesis through a combination of cell-to-cell signalling and cytokine secretion that elicit unique biological effects in progenitors. To understand the molecular signals generated by these niches, we report the generation of caudal hematopoietic embryonic stromal tissue (CHEST) cells from 72-hours post fertilization (hpf) caudal hematopoietic tissue (CHT), the site of embryonic HSPC expansion in fish. CHEST cells are a primary cell line with perivascular endothelial properties that expand hematopoietic cells *in vitro*. Morphological and transcript analysis of these cultures indicates lymphoid, myeloid, and erythroid differentiation, indicating that CHEST cells are a useful tool for identifying molecular signals critical for HSPC proliferation and differentiation in the zebrafish. These findings permit comparison with other temporally and spatially distinct haematopoietic-supportive zebrafish niches, as well as with mammalian haematopoietic-supportive cells to further the understanding of the evolution of the vertebrate hematopoietic system.

Haematopoiesis is an essential process for all vertebrate organisms and is dependent on the generation, homeostasis, and differentiation of hematopoietic stem cells (HSCs). HSCs are tissue-restricted stem cells with the ability to both self-renew and generate lineage-restricted hematopoietic progenitor cells (HPCs)^1^,^2^,^3^,^4^ that eventually differentiate into the multitude of mature blood cells that carry oxygen to distant tissues, prevent blood loss, and fight life-threatening infections. One of the ways that hematopoietic stem and progenitor cells (HSPCs) become developmentally restricted is by receiving instructive signals from their microenvironment. As the developing vertebrate organism has multiple different sites of HSPC emergence, proliferation, and maintenance, understanding the signals produced by these niches is essential^5^,^6^,^7^. Elucidating and modulating the molecular pathways responsible for signalling between the niche and HSPCs that affect proliferation and/or differentiation is critical, because perturbations in blood development and homeostasis directly affect an organisms’ survival.

An increasingly popular model system for studying vertebrate haematopoiesis is zebrafish (*Danio rerio*)^8^. Zebrafish embryos develop externally and are optically transparent. Many hematopoietic lineage-specific transgenic lines exist, allowing the visualization of blood development in real time. Zebrafish are extremely fecund, allowing large scale, whole embryo genetic and small molecule screens for genes and pathways involved in hematopoietic development and disease. Importantly, zebrafish have a strong degree of evolutionary conservation with mammalian hematopoietic development and homeostasis; they have similar mature blood cells^9^, contain *bona fide* HSPCs^10^,^11^,^12^, and have similar genetic control of essential hematopoietic functions^13^. Importantly, drugs discovered in zebrafish show promise for treating human blood disorders^14^.

Zebrafish, like mammals, have shifting sites of haematopoiesis in the developing embryo; HSPCs are generated and expanded temporally in different anatomical regions (for a review, see refs 8,15). To investigate the signals produced by these different hematopoietic sites, we previously generated zebrafish embryonic stromal trunk (ZEST) cells^16^ derived from the site of HSC emergence^17^, which appear to be functionally equivalent to the mammalian aorta-gonad mesonephros (AGM) region. Interestingly, ZEST cells had similar signalling properties to another stromal cell line generated from the kidney^18^, the adult site of haematopoiesis in teleosts that is functionally equivalent to mammalian bone marrow (BM)^19^ in terms of supporting blood haemostasis. While HSCs are generated in the mammalian AGM^20^,^21^ and maintained in the BM^22^, they are transiently expanded in the embryo in the foetal liver (FL)^23^,^24^, which is equivalent to a vascularized region in the developing zebrafish tail referred to as caudal hematopoietic tissue (CHT)^25^. To characterize signalling from this location, we generated a primary stromal line termed caudal hematopoietic embryonic stromal tissue (CHEST) cells. These cells express hematopoietic-supportive cytokines and have endothelial properties. Importantly, CHEST cells also supported HSPC proliferation and differentiation when adult whole kidney marrow (WKM) was plated on them. Examining the signalling properties of these CHEST cells and comparing them to hematopoietic-supportive zebrafish kidney stroma (ZKS) and ZEST cells should illuminate conserved signalling pathways important for hematopoietic support and maintenance. It will also allow the investigation of specific signalling pathways that differ amongst these cells that make these temporally and spatially distinct locations unique. Finally, it will permit comparison of hematopoietic signals in the zebrafish to mammals; these evolutionarily conserved pathways are likely excellent targets to expand blood, generate HSCs, and drive specific lineage differentiation *in vitro*, all of which are important goals for regenerative medicine.

## Materials and Methods

### Zebrafish stocks and embryos

Zebrafish were mated, staged, raised, and maintained in accordance with California State University, Chico (CSU Chico) and University of California, San Diego (UCSD) IACUC guidelines. All experiments were approved by the CSU Chico and UCSD IACUC before being performed. AB wild-type (wt) fish were utilized for these studies.

### Generation of CHEST cells

**Figure 1:**
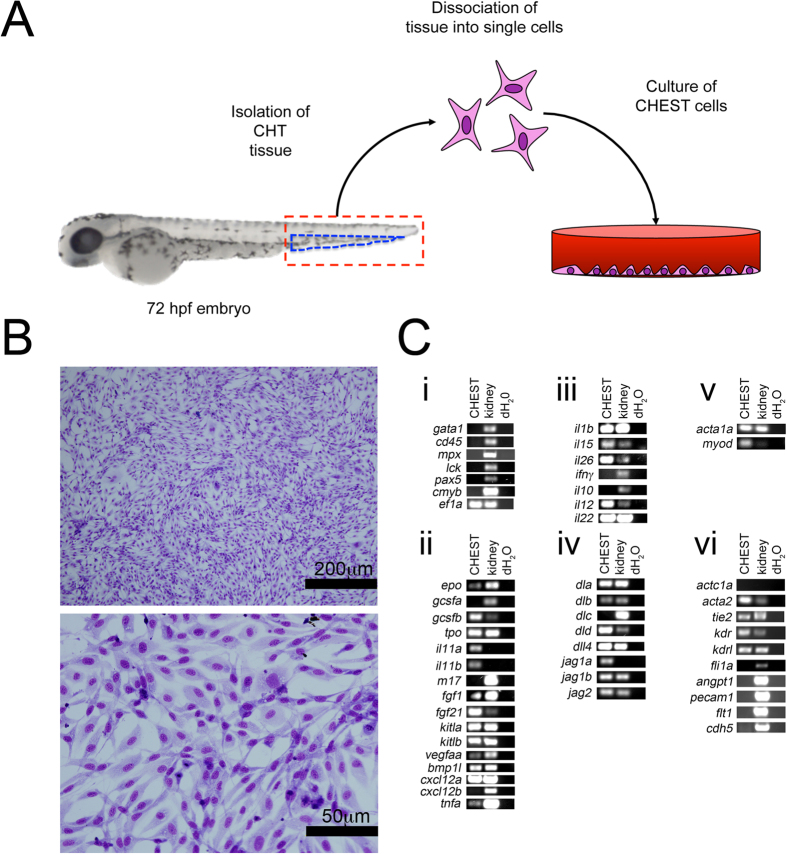
CHEST cells are a primary endothelial-like stromal cell line derived from the CHT of 72hpf zebrafish. (**A**) Isolation and culture strategy for CHEST cells. Red hatched lines indicate the tissue removed for deriving CHEST cells, and the blue hatched lines indicate the anatomical location of the CHT region. (**B**) Monolayers of CHEST cells stained with May-Grünwald Giemsa. Top image: 400x, scale bar is 200 μm; bottom image: 1000x, scale bar is 50 μm. **(C)** RT-PCR analysis of (**i**) hematopoietic, (**ii**) hematopoietic-supportive cytokines, (**iii**) inflammatory signals, (**iv**) Notch pathway mediators, (**v**) skeletal muscle, and (**vi**) cardiac and endothelial transcripts. Gel images have been cropped for clarity of presentation.

CHEST cells were isolated by surgically removing the CHT from the trunk of 72-hour post fertilization (hpf) AB wild type (wt) fish. At 72 hpf, approximately 200 embryos were rinsed three times in sterile embryo medium^26^ in 10 cm^2^ plates. Using an Olympus SZ51 dissecting microscope, the tissue posterior to the yolk tube extension was removed with a sterile scalpel (see Fig. 1A; hatched area denotes the region that was isolated) and finely minced with a surgical scalpel and grown in zebrafish tissue culture medium^16^,^18^ with 5 ng/ml human fibroblast growth factor 2 (FGF2) and 5 ng/ml sodium selenite added in a 12.5 cm^2^ tissue culture flask. The cells that attached to the surface of the flask were grown at 32 °C in 5% CO_2_ until cells achieved ≥ 80% confluence. Cells were trypsinized for 5 minutes and expanded onto 75-cm^2^ tissue culture flasks.

### Morphological characterization of CHEST cells

CHEST cells were grown on glass coverslips in culture media in 24-well tissue culture plates. When cells reached 100% confluence, they were fixed and stained with May-Grünwald Giemsa and visualized by microscopy^27^.

### Proliferation of CHEST cells

CHEST cells were grown in 75 cm^2^ tissue culture flasks and treated with 1) ZKS/ZEST culture media^16^,^18^ alone, 2) ZKS/ZEST culture media with 5 ng/ml FGF2 added, 3) ZKS/ZEST culture media with 5 ng/ml sodium selenite added, or 4) ZKS/ZEST culture media with both FGF2 and sodium selenite. At various time points cells were isolated by trypsinization and enumerated by trypan blue exclusion and counting with a hemacytometer. This method was also utilized to enumerate the difference in growth between ZEST and CHEST cells when they were plated in (1) ZEST media or (2) CHEST media.

### Reverse transcription polymerase chain reaction (RT-PCR) analysis of CHEST cells

RNA was isolated from CHEST cells using a QIAGEN RNeasy kit, and cDNA was generated using the BioRad iScript cDNA synthesis kit. Primers, product sizes, and annealing temperatures used for the RT-PCR characterization of ZEST cells are listed in Table 1.

### Isolation and enumeration of WKM

WKM was isolated as described previously^15^,^27^ and enumerated by trypan blue exclusion and counting with a haemocytometer. To enumerate WKM cells after being cultured on CHEST cells, the stroma was gently rinsed to remove the WKM from the cell monolayers. Cells were concentrated by centrifugation at 300 *g*, cytospun onto slides, stained with May-Grünwald Giemsa, and visualized by microscopy.

### Fluorescence-activated cell sorting (FACS)

WKM was isolated and resuspended in PBS with 0.9% foetal bovine serum. Lymphoid and precursor fractions were sorted and analysed on an Influx high speed sorter (BD Biosciences) by utilizing their unique forward and side scatter characteristics^19^. Sytox red (Life technologies) was used as a cell viability stain.

### Immunofluorescence

CHEST cells were grown on glass coverslips in culture media in 24-well tissue culture plates. When cells reached 100% confluence, they were fixed and stained with 4% paraformaldehyde for 15 min at room temperature, washed with PBS, and left overnight at 4 °C. Slides were permeabilized with PBS with 0.1% Tween-20 (PBT) for 10 min at room temperature, washed with PBS, and blocked with 1% BSA in PBT for 1 hour. Cells were incubated with mouse ZO-1 monoclonal antibody (1:100 dilution; Invitrogen) overnight, washed three times in PBT, incubated with goat-anti-mouse Alexa Fluor 594 secondary (1:200 dilution; Life Technologies) and DAPI (1:1000 dilution), washed three times in PBT, cover slipped, and visualized by fluorescent microscopy^27^.

### Matrigel assays

CHEST cells were plated onto Matrigel precoated 6-well plates (Corning) and incubated at 32 °C for 24 hours in endothelial growth medium-2 (Lonza) followed by brightfield microscopy. After cells were imaged, they were trypsinized, RNA was isolated with a QIAGEN RNeasy kit, and cDNA was generated using the BioRad iScript cDNA synthesis kit for RT-PCR analysis.

### Statistics

Two tailed heteroscedastic student t tests were performed to determine statistical significance.

## Results

### Generation and characterization of hematopoietic-supportive stromal cells

To generate a hematopoietic-supportive stromal cell line, we isolated cells from the caudal hematopoietic tissue (CHT) region of zebrafish, where hematopoietic stem and progenitor cells (HSPCs) expand in early embryonic development. Tissue posterior to the yolk tube extension of 72 hpf fish was surgically removed, finely diced, and plated in tissue-culture flasks with media supplemented with selenium and fibroblast growth factor 2 (FGF2) (Fig. 1A). As with ZKS and ZEST cultures, these cells were grown at 32 °C in a humidified, 5% CO_2_ environment. These caudal hematopoietic embryonic stromal tissue (CHEST) cells divided every 36–48 hours, and grew continuously in culture. We have grown these cells for over 200 passages, and never observed any senescence. Additionally, there was no survival crisis during the early stages of growth, indicating that the cells likely did not derive from a clonal population of cells that survived culture conditions. The cells that grew from this explanted tissue had a stromal morphology (Fig. 1B) when grown on glass coverslips and stained with May-Grünwald Giemsa and appeared homogenous. Importantly, there were no visible hematopoietic cells present in the culture (Fig. 1B, bottom). To confirm that CHEST cells were not contaminated with hematopoietic cells and also to examine if they were spontaneously producing blood, we examined CHEST cells by RT-PCR. *gata1, cd45, mpx, lck, pax5,* and *cmyb* transcripts were not detected in these cultures, indicating that there were no red blood cells, leukocytes, or HSPCs present (Fig. 1Ci), confirming their stromal nature.

To determine if CHEST cells had the capability to support haematopoiesis, we examined their transcript expression by RT-PCR. CHEST cells produce several zebrafish cytokines important for blood cell development including erythropoietin (*epo*^28^), granulocyte colony stimulating factor (*gcsf*^10^,^29^), and thrombopoietin (*tpo*^12^) (Fig. 1Cii). They also expressed hematopoietic supportive cytokines such as *il11a* and *b, fgf1, fgf21, kit* ligands, and *cxcl12a* and *b* (Fig. 1Cii). CHEST cells expressed inflammatory cytokines (Fig. 1Ciii), including *tnfa*, recently implicated in the formation of HSCs^30^. CHEST cells also expressed a multitude of Notch signalling pathway mediators (Fig. 1Civ) and skeletal muscle markers (Fig. 1Cv). Importantly, they also expressed several markers of smooth muscle and endothelial cells, including *acta2, tie2, kdr,* and *kdrl* but not the cardiac-specific muscle marker *actc1a* (Fig. 1Cvi). Together, these data indicated that CHEST cells expressed a multitude of hematopoietic-supportive cytokines, inflammatory molecules, and Notch signalling mediators that would likely support blood development.

**Figure 2:**
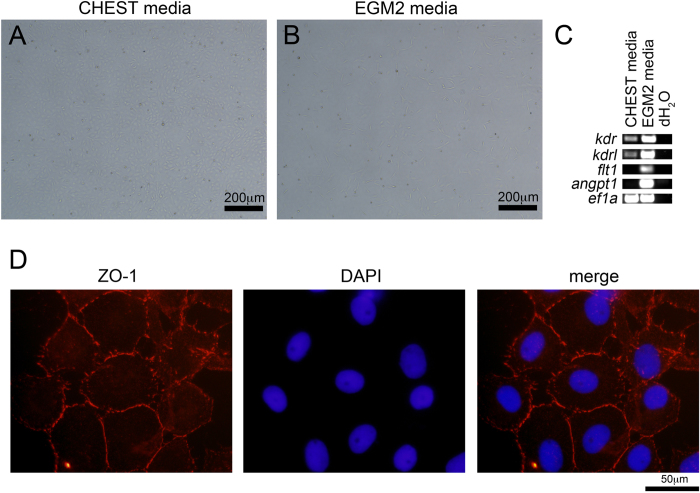
CHEST cells have endothelial-like properties. (**A**) Images of CHEST cells grown on tissue culture plates with CHEST growth media and (**B**) on Matrigel with EGM2 endothelial growth media. Images taken at 200x, scale bar is 200 μm. **(C**) RT-PCR performed on CHEST cells from **(A)** and **(B)** for endothelial-specific transcripts. **(D)** ZO-1 staining (red, left panel), DAPI staining (blue, middle panel), and merged image (right panel) of CHEST cells grown to confluence in CHEST media. Images taken at 1000x, scale bar is 50 μm.

**Figure 3:**
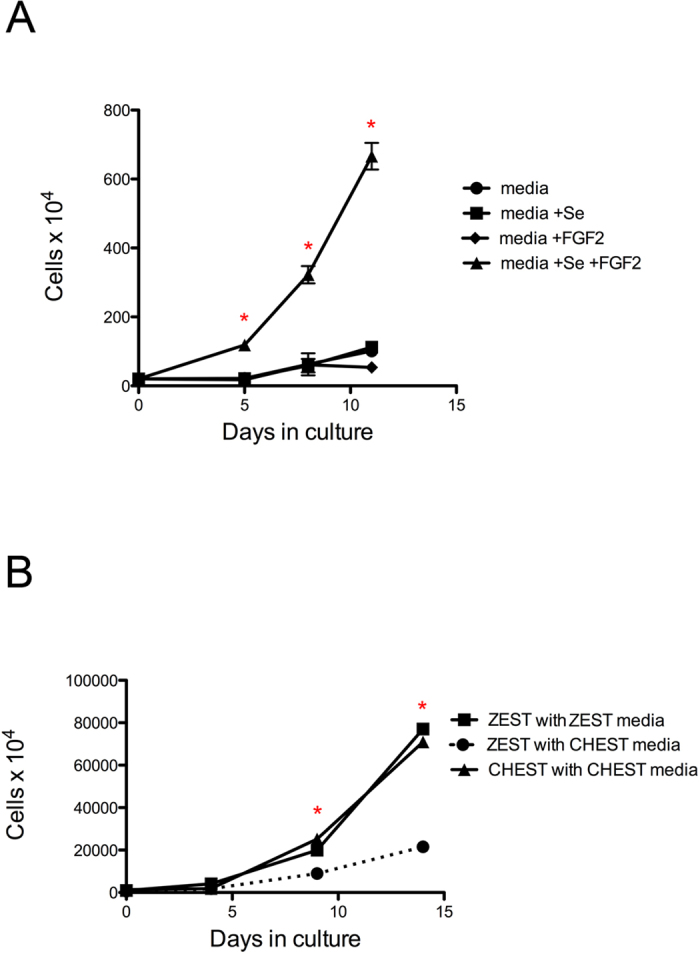
CHEST cells uniquely require selenium and FGF2 for their proliferation. **(A)** CHEST cells were enumerated at various times after culture in media (black circles; n = 3), media with selenium (+Se, black squares; n = 3), media with FGF2 (black diamonds; n = 3), or media with Se and FGF2 (black triangles; n = 3). Error bars denote standard error of the mean. * denotes p < 0.005 when comparing cells grown with CHEST media supplemented with selenium and FGF2 to either of the three other samples. **(B)** ZEST cells were enumerated at various times after culture in ZEST media (black squares; n = 3) and in media with selenium and FGF2 (CHEST media, black circles, dashed line; n = 3). CHEST cells grown in CHEST media (black triangles; n = 3) is shown as a control. Error bars denote standard error of the mean. * denotes p < 0.005 when comparing ZEST cells grown in CHEST media to either of the two other samples.

As CHEST cells expressed several markers of endothelial cells (Fig. 1Cvi), we examined if they would also form capillary networks when plated on Matrigel-coated plates with endothelial growth media-2 (EGM2), which is a capability of cells with endothelial potential^31^,^32^,^33^. When CHEST cells were plated on standard tissue culture plates in CHEST media, no branching activity after 24 hours was observed (Fig. 2A). However, when plated on Matrigel in EGM2 media, cellular elongation, a property of endothelial-like cells, was observed (Fig. 2B). To further examine the cells’ endothelial-like nature, we harvested them after 24 hours in culture and performed RT-PCR for *kdr, kdrl, flt1,* and *angpt1*, all of which were upregulated in endothelial growth conditions (Fig. 2C). To examine if CHEST cells would form tight junctions *in vitro*, a characteristic of confluent endothelial monolayers^34^ and an essential component of angiogenesis and barrier formation of primary endothelial cells^35^, immunoblotting with a monoclonal antibody directed against zona occludens 1 (ZO-1) was performed, indicating that CHEST cells did form tight junctions between one another when grown at high confluence in culture (Fig. 2D). CHEST cells also had unique growth properties in different media formulations. CHEST cells do not proliferate well in ZKS/ZEST media^16^,^18^ (Fig. 3A), and multiple attempts to derive them in this media failed (data not shown). Prior studies culturing zebrafish endothelial stromal cells added selenium and FGF2 to the culture media^31^, so we also added these supplements. Media with either selenium or FGF2 added did not significantly increase the growth rate of CHEST cells. Instead, both additives were required for optimal growth (Fig. 3A). Importantly, the combinatorial addition of selenium and FGF2 is not universally supportive of stromal cell lines; growing ZEST cells in this media did not support ZEST cell expansion as well as CHEST cell expansion (Fig. 3B). Together, these RT-PCR, immunohistochemical staining, and growth condition data indicate that CHEST cells have endothelial-like properties and are different types of stromal cells than previously derived embryonic primary stromal cell lines.

### CHEST cells encourage proliferation of hematopoietic cells *in vitro*

**Figure 4:**
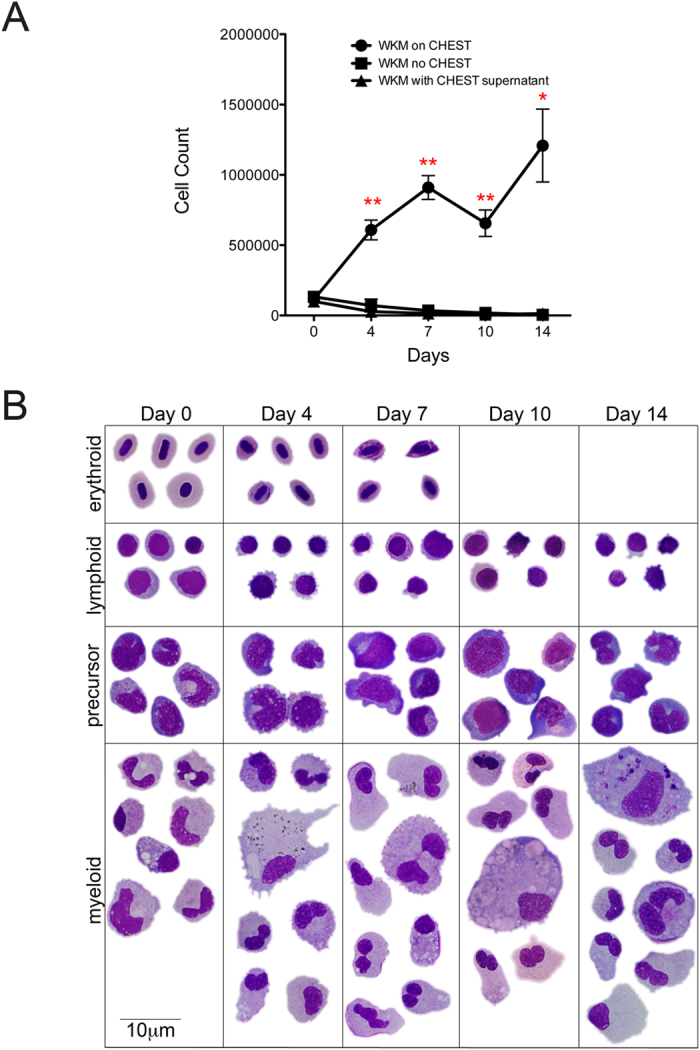
CHEST cells support hematopoietic expansion. (**A**) WKM from adult zebrafish was plated on CHEST cells and enumerated at various times (black circles; n = 8), plated in culture with no stromal under layer (black squares; n = 6), or plated with no CHEST cells in CHEST cell supernatant media (black triangles; n = 3). Error bars denote standard error of the mean. * denotes p < 0.005 and ** denotes p < 0.0005 when comparing cells grown on CHEST cells to either of the two other samples. (**B**) Composite May-Grünwald Giemsa images of hematopoietic cells isolated from the cultures (1000x, scale bar is 10 μm).

To determine if CHEST cells would support haematopoiesis and the expansion of zebrafish blood cells, we isolated WKM from the kidney, which is the main site of adult teleost haematopoiesis. WKM was plated on CHEST cells that were 80% confluent, and cell counts and cytocentrifuge preparations were performed at days 4, 7, 10, and 14 of culture. Cells plated on CHEST stroma increased over two weeks, while WKM with no stromal support died off during the same time period (Fig. 4A). To confirm that the cells that proliferated were hematopoietic, we also cytocentrifuged the samples at each time point and stained them with May Grünwald-Giemsa. At each time point, cells with lymphoid, precursor, and myeloid morphologies were identified (Fig. 4B and Table 2). Mature red blood cells were identified up to day 7 in culture, likely due to a lack of exogenous fish transferrin being added to the culture; this lack of mature red blood cell maintenance was not surprising, and was also seen in ZKS and ZEST cells^16^,^18^. As with ZKS and ZEST cells, the support of haematopoiesis was dependent on cell-cell contact; simply removing CHEST cell conditioned media and adding it to WKM was not enough to support their survival (Fig. 4A). To ensure that the CHEST cells were not a mixed population of cells with varying hematopoietic-supportive qualities, a limiting dilution of the original CHEST culture generated ten different clonal lines. All of these clones expanded WKM numbers in a similar manner (data not shown), indicating that the CHEST cells are homogenous and not comprised of mixed cell populations that have variable hematopoietic supportive qualities. Overall, these data indicate that CHEST cells support the proliferation and maintenance of adult zebrafish hematopoietic cells.

### CHEST cells support the proliferation and differentiation of HSPCs

**Figure 5:**
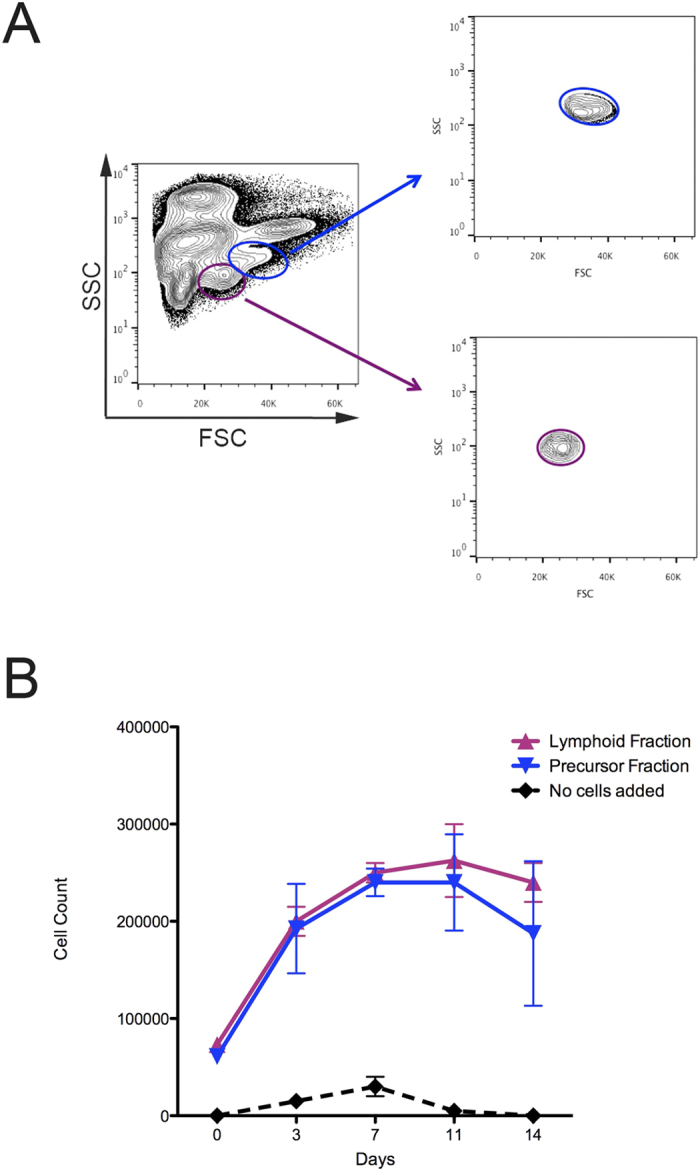
CHEST cells expand HSPCs in culture. (**A**) FACS isolation schematic. Precursor fraction (blue) isolated from WKM (left panel) was reanalysed after sorting and deemed to be over 95% pure (right panel). Lymphoid fraction (purple) isolated from WKM (left panel) was reanalysed after sorting and deemed to be over 95% pure (right panel). **(B)** Cultures started from the lymphoid fraction (purple triangles, n = 3) and precursor fraction (blue triangles, n = 3) were enumerated over time in culture on CHEST cells. Cultures with no fractions added were also counted to ensure that CHEST cells were not spontaneously generating hematopoietic cells (black diamonds, n = 3).

**Figure 6:**
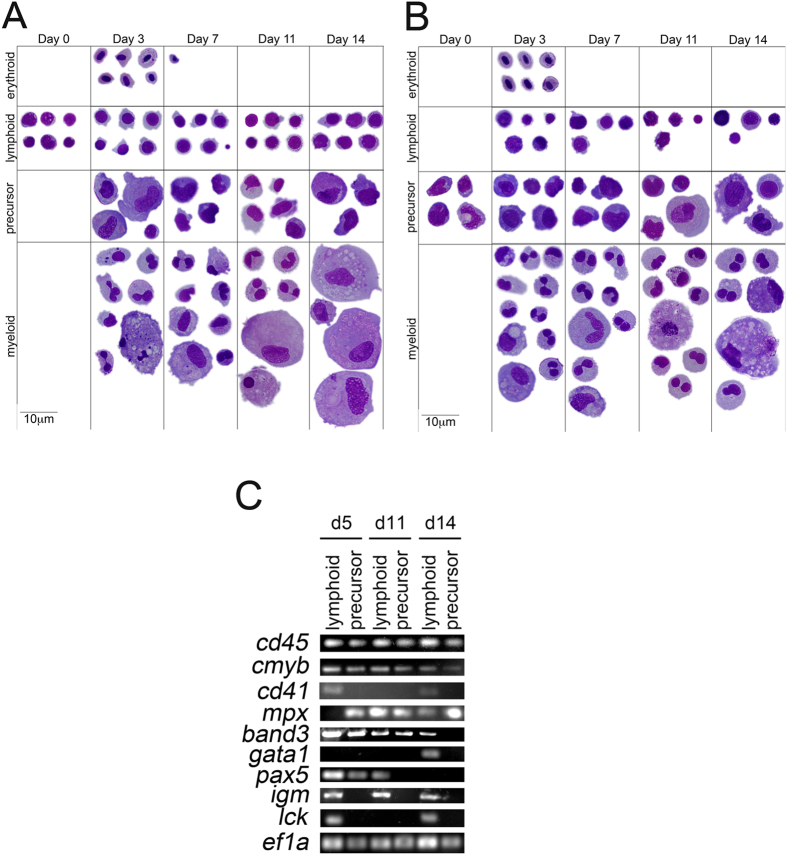
CHEST cells encourage differentiation of HSPCs in culture. (**A**) Composite May-Grünwald Giemsa images of hematopoietic cells isolated from the lymphoid fraction in Fig. 5 (1000x, scale bar is 10 μm). **(B)** Composite May-Grünwald Giemsa images of hematopoietic cells isolated from the precursor fraction in Fig. 5 (1000x, scale bar is 10 μm). **(C)** RT-PCR of leukocyte- (*cd45*), progenitor- (*cmyb*), erythromyeloid- (*cd41, mpx, band3*, and *gata1*), B cell- (*pax5* and *igm*), and T cell- (*lck*) specific transcripts isolated from the cultures as in **A** and **B**. Gel images have been cropped for clarity of presentation.

To determine if CHEST cells could also encourage the proliferation and differentiation of HSPCs, the lymphoid (Fig. 5A, purple line) and precursor (Fig. 5A, blue line) fractions of adult WKM were isolated by FACS. The lymphoid population contains not only mature lymphoid cells, but also HSPCs and HSCs, while the precursor population contains HSPCs that can give rise to erythroid, myeloid, and lymphoid cell populations. These populations were validated to be over 95% pure (Fig. 5A), after which they were plated on top of 80% confluent CHEST cells. At days 3, 7, 11, and 14, cell counts were performed, indicating that both populations were expanding on CHEST stroma (Fig. 5B). To confirm that these progenitor populations were also differentiating into mature erythroid, lymphoid, and myeloid populations, cells were cytocentrifuged at each time point. The lymphoid cell population maintained lymphoid cells throughout two weeks, but also differentiated into more mature precursor cells and finally into erythrocytes and myeloid cells (Fig. 6A and Table 2). In a similar fashion, the precursor population generated mature erythroid, lymphoid, and myeloid cells over the two-week experiment (Fig. 6B). To confirm the expression of mature cell markers during the differentiation time course, another experiment was performed and cells were isolated at days 5, 11, and 14 and subjected to RT-PCR. In each population, *cd45*^+^ leukocytes were detected, as well as cells expressing *cmyb*, a marker of HSPCs (Fig. 6C). All populations also expressed erythromyeloid markers such as *cd41, mpx, band3*, or *gata1. igm*^+^ mature B cells were only seen in lymphoid populations, as were *lck*^+^ T cells. While *pax5*, an early marker of B cell differentiation was seen in the day 5 precursor population, this marker was not seen at later time points of the experiment. Together, these data indicate that CHEST cells expanded and differentiated a multitude of hematopoietic cell types over two weeks. While all cell types were observed morphologically, RT-PCR and differential counts data indicate that CHEST cells favor the differentiation and proliferation of myeloid cells over the two-week experimental period.

## Discussion

Zebrafish have been utilized extensively to study haematopoiesis: they develop externally and are optically transparent, allowing the visualization of blood formation. Importantly, zebrafish are fecund and genetically amenable, allowing large-scale mutagenesis and chemical screens for elucidating genes and molecular pathways involved in haematopoiesis to be performed. As zebrafish share a multitude of hematopoietic-supportive molecular pathways with other vertebrates (reviewed in ref. 13), findings in the zebrafish have utility for understanding human disease. However, while forward genetic screens have been utilized to identify genes essential for the generation of primitive blood and the emergence of HSCs, they have not elucidated genes essential for HSPC proliferation and differentiation due to a lack of methodologies to functionally assess them. To address this caveat we previously generated cell lines from the adult zebrafish kidney (ZKS cells^18^) and from the site of HSC emergence in the developing embryo (ZEST cells^16^), which allowed us to develop clonal methylcellulose assays to examine cytokine signalling and HSPC differentiation^10^,^11^,^12^. While ZKS and ZEST cells have proven effective as a way to develop and discover new cytokines involved in haematopoiesis, they are not the only hematopoietic niches that exist in the developing zebrafish. In our quest to generate multiple hematopoietic-supportive cell lines, we also isolated and cultured CHEST cells.

CHEST cells are of interest because of the unique hematopoietic niche that they are isolated from. First described over 10 years ago, the CHT is a transient anatomical location surrounding a plexus comprised of endothelial and fibroblastic reticular cells^25^. This location is similar to the murine FL, in that HSCs that extravasate from the dorsal aorta travel to and become lodged in the CHT plexus to expand their numbers. From there, these cells emigrate to either seed the kidney, which is the main site of adult haematopoiesis in the zebrafish, or they travel to the thymus to differentiate into T cells. Recent work indicates that the plexus of the CHT is dynamic, and that when HSPCs lodge there, perivascular endothelial cells remodel so that they surround the HSPCs to form a stem cell “pocket^36^.” Referred to as “endothelial cuddling,” this behaviour has also been visualized in the mouse FL^36^.

Interestingly, CHEST cells share many qualities with the perivascular cells described in these previous reports, indicating that they are likely perivascular in nature. To visualize “endothelial cuddling,” Tamplin *et al*. utilized a transgenic zebrafish whereby the promoter of *cxcl12a* drove DsRed fluorescence^36^; CHEST cells express this important chemokine. CHEST cells also express *kitlgb*, a chemokine expressed in the CHT and required for HSPC expansion in this region^37^. Importantly, CXCL12 and KITLG are known HSPC-supportive factors produced in the stromal perivascular niche of mammalian bone marrow^38^,^39^,^40^. Recent findings indicate that the CHT region expresses *tpo* and *gcsf*^37^, which are cytokines essential for HSPC proliferation^10^,^12^ that are also generated by CHEST cells. Like CHEST cells, perivascular cells also produce VEGF which stimulates endothelial cell survival^41^. Based on the tight binding between the cells that comprise the vascular niche of the CHT^36^, it is not surprising that CHEST cells express ZO-1, a central regulator of endothelial junctions that regulates cell migration and barrier formation^35^. Finally, CHEST cells elongate when plated on Matrigel coated plates, upregulating and expressing endothelial transcripts in these conditions, indicating their perivascular nature.

Although CHEST cells support haematopoiesis *in vitro*, they are distinct from other hematopoietic-supportive stromal cell lines that we have isolated and characterized. First, they are dependent on FGF2 and selenium present in the growth media, which slow the growth of ZEST cells. FGF2 and selenium were required for the generation of other zebrafish endothelial cell lines^31^,^42^, and have been implicated in angiogenesis^43^, HSPC expansion^44^, and cytokine-induced expression of adhesion molecules on endothelial cells^45^,^46^. When we tried to derive CHEST cells without FGF2 and selenium we were unsuccessful. Additionally, differentiating hematopoietic cells on CHEST without these media supplements proved fruitless. While CHEST cells express similar hematopoietic-supportive cytokine transcripts when compared to ZKS and ZEST cells, CHEST cells also express endothelial-specific markers such as *tie2, kdr, kdrl,* and *vegfaa*. One of the most striking differences between CHEST, ZKS, and ZEST cells is that CHEST cells seem to weakly direct mature lymphoid differentiation. While lymphoid cells could survive over a two-week incubation time, and were detected morphologically when we plated HSPCs onto CHEST cells, no definitive lymphoid cell markers were detected. This is in agreement with studies that indicate cellular immigrants to the thymus that left the CHT did not express *rag1*^25^; these cells did not differentiate in the CHT, but needed to mature in the thymus. Likewise, mature B cells are not seen in the zebrafish embryo until 3 weeks post-fertilization^47^, but the CHT is only hematopoietic until 2 weeks post-fertilization^25^. We believe that this is likely due to the fact that zebrafish HSCs and HSPCs have morphological characteristics of lymphoid cells^15^,^19^; when isolated and stained they look very similar. They even have the same light scatter and granularity as lymphoid cells^19^, preventing easy isolation/identification from B and T cells with FACS. In essence, even though we observed cells that were morphologically “lymphoid” in our differentiation assays, they were likely HSPCs, as these cell types have similar appearances. This is further bolstered by the expression of *cmyb* throughout the experiment, while no definitive B and T cell transcripts were detected. In the future, it will be of interest to transplant these “lymphoid” cells back into zebrafish and show short-term or long-term engraftment, which is the gold standard for proving HSPC or HSC identity, respectively.

In the future, it will be of interest to compare the transcriptome of CHEST cells to other hematopoietic-supportive cell lines in the zebrafish^16^,^18^ to determine what signals are shared amongst these cells, and what signals are unique. It will also be of interest to compare the signalling properties to thymic epithelium, the site of T cell differentiation, to see what properties exist in these distinct tissues that support HSPCs differentiating into mature lymphoid cells. Finally, it would be useful to compare these to other hematopoietic-supportive stromal cell lines and perivascular-derived mesenchymal stromal cell lines previously generated^31^,^42^. The goal of all of these studies would be to eventually compare the transcriptome of these zebrafish cell lines to the mammalian sites of haematopoiesis. Investigations have elucidated a core molecular network of mRNA transcripts required for HSPC support in murine cell lines^48^, and it would be of interest to compare the expression of genes shared amongst vertebrate phyla involved in HSPC support. Undoubtedly these shared transcripts will shed light on genes essential for the process of HSPC generation, proliferation, and differentiation, as they have likely been conserved over the past 400+ million years of vertebrate evolution.

## Additional Information

**How to cite this article:** Wolf, A. *et al*. Zebrafish Caudal Haematopoietic Embryonic Stromal Tissue (CHEST) Cells Support Haematopoiesis. *Sci. Rep.* **7**, 44644; doi: 10.1038/srep44644 (2017).

**Publisher's note:** Springer Nature remains neutral with regard to jurisdictional claims in published maps and institutional affiliations.

## Figures and Tables

**Table 1:** Primer sets used for RT-PCR characterization of CHEST cells.

Gene	Definition	Forward primer (5′-3′)	Reverse primer (5′-3′)	Annealing temperature (°C)	Product size (bp)
*tie2 (tek)*	TEK tyrosine kinase, endothelial	GAAGTGTGTGTGTGCAAAAG	CAGTGTTGATCTCCACATCC	55	134
*kdr*	kinase insert domain receptor	TCAGACTCAGAGCTCTTGAG	GACTATAGTAGGGGTCGGTG	55	122
*tpo (thpo*)	thrombopoietin	ATGGAAAGCAACGTGACTGG	CCACTGCAGATTACCTTTTC	55	178
*tnfa*	tumor necrosis factor a	GAGAGATCGCATTTCACAAG	TTCCTCAGTCAGTTCAGACG	55	230
*kdrl*^31^	kinase insert domain receptor like	GCCTCGGGTCAATGCTGTTCC	GTCCGGTTGCCAAGTTCATTCC	60	561
*flt1*^31^	fms-related tyrosine kinase 1 (vascular endothelial growth factor/vascular permeability factor receptor)	ATCCGCAGTGTTATATTCCTCCAT	TTGCGCTCCTTTTGCCGATACCT	60	410
*angpt1*^31^	angiopoietin 1	CTACAACAGAGCGCCGTCCAC	AGGTCCTGCTGTCTCTGAAG	60	419
*fli1a*	fli-1 proto-oncogene, ETS transcription factor a	ATGGACGGAACTATTAAGGAGGC	TTAGTAGTAACTACCAAGGTGTG	60	1355
*pecam1*^31^	platelet/endothelial cell adhesion molecule 1	GACGGGCACGCTGATGTTTCTCTTC	CTGCACGCTCCCTTTCTCGGTTTTG	60	427
*cdh5*^31^	cadherin 5	CCCCGTTTTCGATTCTGACC	CTTTGAGGCTTAGCATTCCATCTT	60	515
*acta1a*^16^	actin, alpha 1a, skeletal muscle	GAAAAGAGCTACGAGCTTCC	GTAAGTGGTCTCGTGAATGC	50	129
*myod1*^16^	myogenic differentiation 1	ATGGCATGATGGATTTTATG	TTTATTATTCCGTGCGTCAG	50	107
*actc1a*^16^	actin, alpha, cardiac muscle 1a	TGCTGTCTTTCCCTCTATTG	GAGTGAGGATACCCCTCTTG	50	116
*acta2*^16^	actin, alpha 2, smooth muscle, aorta	TGGATCTGGACTGTGTAAGG	ACTATCTTTCTGCCCCATTC	50	121
*gata1a*^49^	GATA binding protein 1a	TGAATGTGTGAATTGTGGTG	ATTGCGTCTCCATAGTGTTG	55	650
*cd45 (ptprc*)^49^	protein tyrosine phosphatase, receptor type, C	AGTTCCTGAAATGGAAAAGC	GCACAGAAAAGTCCAGTACG	55	140
*ef1a (eef1a1l1*)^49^	eukaryotic translation elongation factor 1 alpha 1, like 1	GAGAAGTTCGAGAAGGAAGC	CGTAGTATTTGCTGGTCTCG	55	123
*epo*^18^	erythropoietin	ACTTGTAAGGACGATTGCAG	TATCTGTAATGAGCCGATGG	55	156
*gcsfa (csf3*)^10^	colony stimulating factor 3a, granulocyte colony stimulating factor	AACTACATCTGAACCTCCTG	GACTGCTCTTCTGATGTCTG	55	165
*gcsfb (csf3b*)^10^	colony stimulating factor 3b, granulocyte colony stimulating factor b	GGAGCTCTGCGCACCCAACA	GGCAGGGCTCCAGCAGCTTC	55	184
*il-11a*	interleukin 11a	GACAAGCTGAGCAATCAGAC	GGAGCTGAGAAAGAGTAGGC	50	172
*il-11b*	interleukin 11b	TTGAACATTCGCTATCATCC	GAGTAATCGTTCCCCAATTC	50	166
*m17*^16^	il-6 subfamily cytokine M17	CTTGATTGCCGTTCAGTTAG	TGACCGGAGATTGTAGACAC	50	210
*fgf1*^18^	fibroblast growth factor 1	ATACTGCGCATAAAAGCAAC	AGTGGTTTTCCTCCATCTTC	50	154
*fgf21*^18^	fibroblast growth factor 21	CGGTGGTGTATGTATGTTCC	GTAGCTGCACTCTGGATGAC	50	203
*kitlga*^18^	kit ligand a	GGATTCAATGCTTGACTTTG	TGTACTATGTTGCGCTGATG	50	205
*kitlgb*^16^	kit ligand b	GGCAACCAGTCCACCAATAAG	CACTTTTCCCTTCTGTAGTGGC	50	135
*vegfaa*^16^	vascular endothelial growth factor Aa	GAAACGTCACTATGGAGGTG	TTCTTTGCTTTGACTTCTGC	50	121
*bmp1l*^18^	bone morphogenic protein 1, like	GGATGGATATTGGAGGAAAG	CTTTGTTCGGTCTGTAATCG	50	230
*cxcl12a*^16^	chemokine (C-X-C motif) 12a, stromal cell-derived factor 1a	CGCCATTCATGCACCGATTTC	GGTGGGCTGTCAGATTTCCTTGTC	50	297
*cxcl12b*^16^	chemokine (C-X-C motif) 12b, stromal cell-derived factor 1b	CGCCTTCTGGAGCCCAGAGA	AGAGATTCTCCGCTGTCCTCC	50	291
*il-1b*^16^	interleukin 1, beta	TCCACATCTCGTACTCAAGG	CAGCTCGAAGTTAATGATGC	50	227
*ifnɣ*^16^	interferon, gamma 1-2	TACATAATGCACACCCCATC	TCCTTTGTAGCTTCATCCAC	55	158
*il-26*^18^	interleukin 26	TGAAAAGATGTGGGATGAAC	ACTGATCCACAGCAAAACAC	55	214
*il-15*^18^	interleukin 15, like	CCAAGTCCACAATTACATGC	TCTTTGTAGAGCTCGCAGAC	55	166
*il-12a*^18^	interleukin 12a	GTGAGTCTGCTGAAGGAGTG	AGTGACATCATTTCCTGTGC	50	167
*il-1018*	interleukin 10	ATGAATCCAACGATGACTTG	TCTTGCATTTCACCATATCC	50	222
*il-22*	interleukin 22	CTACCTGCGATATGAAGTGC	GAAATATGGAAGCAGTCGTG	50	171
*dla*^18^	δA	ACGACGATTTGAGTATGACG	GGGATTGGCACTTTATATCC	50	186
*dlb*^18^	δB	TTCCGTGTTTAATGATTTGG	CACTCCACAGAAACTCTTGC	50	158
*dlc*^18^	δC	TGGTGGACTACAATCTGAGC	ACCTCAGTAGCAAACACACG	50	169
*dld*^16^	δD	AACCCAGACCGTCTGATCAGT	CCGGGTTTGTCGCAAAAGCCA	50	308
*dll4*^18^	δ-like 4	CTCTTTCAGCACACCAATTC	TGAACATCCTGAGACCATTC	50	189
*jag1a*^18^	jagged 1a	TGATTGGTGGATACTTCTGC	AATCCATTGAGTGTGTCCTG	55	238
*jag1b*^18^	jagged 1b	CTGTGAGCCATCTTCTTCAG	AGCAAAGGAACCAGGTAGTC	55	213
*jag2*^18^	jagged 2	AATGACTGTGTGAGCAATCC	GTCATTGACCAGATCCACAC	50	174
*mpx*^50^	myeloid-specific peroxidase	TGATGTTTGGTTAGGAGGTG	GAGCTGTTTTCTGTTTGGTG	55	158
*pax5*	paired box 5	GACCCTCTCCAGGATTCTCC	TGGGGTTTGACCAGGAAATA	60	501
*lck*	LCK proto-oncogene, Src family tyrosine kinase	GCCAGAGGGGTTACATACCA	TGATAGCCACTTGACGGTTG	60	501
*cmyb (myb*)	v-myb avian myeloblastosis viral oncogene homolog	AAGACCAACGGGTGATTGAG	CAGTGCCATGTTGTGGAAAG	60	507
*cd41 (itga2b*)^49^	Integrin, alpha 2b	CTGAAGGCAGTAACGTCAAC	TCCTTCTTCTGACCACACAC	55	197
*band3 (slc4a1a*)	solute carrier family 4 (anion exchanger), member 1a (Diego blood group)	CAACTTCCAGAAGAAGATG	TTGACCATACCACCAAATG	55	144
*igm (ighm*)^50^	immunoglobulin heavy constant mu	AGCTTCTCTAGCTCCACCAG	ATTTTGGTGAAATGGAATTG	55	477

**Table 2:** Differential counts of haematopoietic differentiation on CHEST cells.

Days in culture	Cells plated on CHEST	Cell types counted after culture on CHEST cells				
		Erythroid	Lymphoid	Precursor	Myeloid	Total cells
0	WKM	65 (44.2%)	14 (9.5%)	20 (13.6%)	48 (32.7%)	147
	Precursor fraction	1 (0.5%)	7 (3.4%)	195 (94.2%)	4 (1.9%)	207
	Lymphoid fraction	2 (1.3%)	150 (96.8%)	3 (1.9%)	0 (0%)	155
3	WKM	186 (22.1%)	122 (14.5%)	131 (15.6%)	402 (47.9%)	840
	Precursor fraction	19 (6.1%)	13 (4.2%)	75 (24%)	205 (65.7%)	312
	Lymphoid fraction	11 (3.4%)	78 (24.5%)	82 (25.7%)	148 (46.4%)	319
7	WKM	39 (6%)	86 (13.2%)	95 (14.5%)	433 (66.3%)	653
	Precursor fraction	2 (1%)	18 (8.8%)	53 (26%)	131 (64.2%)	204
	Lymphoid fraction	5 (3%)	46 (27.2%)	24 (14.2%)	94 (55.6%)	169
11	WKM	18 (3.4%)	76 (14.2%)	112 (21%)	331 (61.6%)	537
	Precursor fraction	1 (0.5%)	22 (10.9%)	26 (12.9%)	150 (74.6%)	201
	Lymphoid fraction	1 (0.7%)	41 (28.9%)	9 (6.3%)	91 (64.1%)	142
14	WKM	2 (0.7%)	42 (14.1%)	68 (22.8%)	186 (62.4%)	298
	Precursor fraction	1 (0.6%)	20 (11.7%)	19 (11.1%)	131 (76.6%)	171
	Lymphoid fraction	0 (0%)	48 (38.1%)	16 (12.7%)	62 (49.2%)	126
